# Physiological functional traits explain morphological variation of *Ulva prolifera* during the drifting of green tides

**DOI:** 10.1002/ece3.8504

**Published:** 2022-01-17

**Authors:** Chen Guan, Xinyu Zhao, Tongfei Qu, Yi Zhong, Chengzong Hou, Zhihao Lin, Jinhui Xu, Xuexi Tang, Ying Wang

**Affiliations:** ^1^ College of Marine Life Sciences Ocean University of China Qingdao China; ^2^ Laboratory for Marine Ecology and Environmental Science Qingdao National Laboratory for Marine Science and Technology Qingdao China

**Keywords:** morphological variation, photosynthetic system, reproductive allocation, surface area to volume ratio, *Ulva prolifera*

## Abstract

*Ulva prolifera* green tides, one of the greatest marine ecological disasters, originate in the southern Yellow Sea of China and obtain the highest biomass in Haizhou Bay (latitude around 35° N) during northward drift. *U*. *prolifera* shows different morphologies from southern Haizhou Bay (SH) to northern Haizhou Bay (NH). Owing to the distinct nutrient environments between SH and NH, we hypothesized that thalli in NH with poor nutrients increased the surface area to volume ratio (SA:VOL) to better absorb nutrients. Here, we tested this hypothesis by comparing the SA:VOL of thalli in SH and NH. The results showed that the thalli in NH had a lower SA:VOL than those in SH, and SA:VOL had positive relationships with temperature and nutrients, contrary to the general hypothesis. The novel results suggested that morphological differences of *U*. *prolifera* were the result of developmental state rather than environmental acclimation. Indicators of reproduction (reproductive allocation ratio) were negatively related to variation in tissue contents of C, N, P, and crude protein, whereas indicators of growth (tissue contents of C, N, P, and crude protein) showed significant positive influences on SA:VOL. The results indicated that a trade‐off relationship between reproduction and growth existed in the northward drift. All the results suggested that physiological functional traits affected morphological variation of *U*. *prolifera* in different environmental conditions during the drifting of green tides. This study presents new insights into the opportunist species nature of *U*. *prolifera* through morphological variation and associated functional consequences.

## Graphical abstract

This manuscript highlighted a trade‐off relationship between growth and reproduction existed in the northward drift of green tides caused by *U. prolifera*, and all the results suggested that physiological functional traits affected morphological variation of *U. prolifera* in different environmental conditions during the drifting. Principal findings of the manuscript: (1) previous studies demonstrated that large‐scale green tides, mainly the genus *Ulva prolifera*, have occurred in the Yellow Sea of China since 2007 and obtain the highest biomass in Haizhou Bay. *U. prolifera* displayed distinct morphological differences during the drift northward. In order to find the reason about morphological variation of *U. prolifera*, we hypothesized that the thalli of *U. prolifera* adapt to the environmental changes by the variation in morphology to enhance nutrient uptake in northern Haizhou Bay (NH), an area with relatively less nutrients. (2) However, by comparing the surface area to volume ratio (SA:VOL), we found that the thalli in NH had a lower SA:VOL than those in SH, and SA:VOL had positive relationships with temperature and nutrients, contrary to the general hypothesis. (3) The novel results suggested that morphological differences of *U. prolifera* were the result of developmental state rather than environmental acclimation. Indicators of growth (Fv/Fm, Y(II)) and relative growth rate showed positive correlations with SA:VOL, whereas indicators of reproduction (reproductive allocation ratio) exhibited an opposite result and were negatively related to variation in tissue contents of C, N, P, and crude protein.

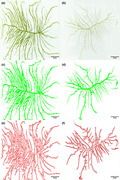

## 1 INTRODUCTION

Green tides caused by excessive growth of green algae, mainly the genus *Ulva*, are a common type of harmful algal bloom (Anderson et al., 2002; Fletcher, 1996). Over the past several decades, green tides have been reported worldwide, especially in areas where eutrophication has been increasing (Anderson et al., 2002; Bäck et al., 2000). Large‐scale green tides in the southern Yellow Sea have been recurring for 14 consecutive years since 2007. The algae that make up the green tides in the Yellow Sea were identified as the opportunist species *Ulva prolifera* (Wang et al., 2015; Ye et al., 2008; Zhang et al., 2019). Previous studies and satellite remote‐sensing data showed that *U*. *prolifera* green tides originated from offshore areas of Jiangsu province, bloomed in the Yellow Sea, and declined in offshore areas of Shandong province (Bao et al., 2015; Hu et al., 2010; Huo et al., 2016; Wang et al., 2015).

Environmental conditions are significantly different during the northward drifting of green tides (Zhang, Su, et al., 2020). Nutrient enrichment has occurred in most of the coastal waters in the southern Yellow Sea over the last decade; the contents of dissolved inorganic nitrogen (DIN) and phosphate–phosphorus (PO_4_^3−^‐P) have increased significantly (State Oceanic Administration, 2007–2019). Therefore, the southern Yellow Sea has long been in a state of eutrophication (Zhang, Wang, et al., 2020). Affected by terrestrial rivers, the distribution of nutrients in the southern Yellow Sea usually shows a decreasing trend from the coast to the ocean (Shi et al., 2015). During the northward drift of *U*. *prolifera*, especially in Haizhou Bay (latitude around 35°N), there are significant differences in DIN and PO_4_^3−^‐P contents (Wang et al., 2018). DIN and PO_4_^3−^‐P south of 35°N were significantly higher than those north of 35°N (Guan et al., 2021). Locations south of 35°N provided 96% of the nitrogen and 87% of the phosphorus for the development of green tides (Zhang, Su, et al., 2020). Meanwhile, latitudes around 35°N in the Yellow Sea generated approximately 80% of the green tide biomass (Zhang, Wang, et al., 2020).

Macroalgae show varied growth responses to different environmental factors (Angell et al., 2015; Dawes et al., 1978). The relationship between nutrients and algae has attracted the attention of several researchers (Kamer et al., 2001; Liu et al., 2013; Wang et al., 2018). Nutrients have been identified as the key factors affecting the formation of the green tides (Wang et al., 2015). The concentrations and forms of nutrients have important influence on the biomass and species composition of phytoplankton (Justić et al., 1995; Tyrrell, 1999). N‐limited microalgae have profound effects on respiratory and photosynthetic metabolism (Turpin et al., 1988). Elevated temperatures are expected to increase eutrophication, further promoting the growth of filamentous species, especially green algae (Takolander et al., 2017). Compared with *U*. *compressa*, *U*. *flexuosa*, and *U*. *linza*, *U*. *prolifera* demonstrated greater tolerance to high temperatures and light intensity (Cui et al., 2015). *Ulva* species have a broad range of tolerance to the environmental conditions of irradiance, temperature, salinity, and N and P nutrients (Taylor et al., 2001; Wu et al., 2000). The thalli of *Ulva* display mechanisms that enable adaptation to diverse types of stress conditions (Mou et al., 2013; Wang et al., 2012; Zou et al., 2007). Zhao et al. (2019) found that NPQ, CEF, and energy redistribution between PSI and PSII all played important roles in the strong photosynthetic plasticity of *U*. *prolifera* during changes of the in‐situ environment (Zhao et al., 2019). PSI‐driven cyclic electron flow allowed intertidal *Ulva* to survive in desiccated conditions (Gao et al., 2011).

Previous study has demonstrated that the morphological plasticity of macroalgae reflects local environmental conditions. Aberrant growth forms were believed to have better potential for survival in the prevailing conditions (Valiela et al., 1997). The morphological features of *Ulva* were significantly related to salinity and nutrient concentrations, and both phosphate and nitrite concentrations were positively correlated with thallus length (Messyasz & Rybak, 2011). Moreover, the same species of *Ulva* may show different morphologies. Malta et al. (1999) found that one highly polymorphic species of *Ulva* occurred in three different morphologies in the eutrophic brackish “Veerse Meer” lagoon (Malta et al., 1999). Similarly, monostromatic sheets have been found in *U*. *prolifera*, reproducing only by cell regeneration into typical tubular thalli in eutrophic marine environments (Blomster et al., 2002). In addition, *U*. *prolifera* also displayed four morphological forms, filamentous, tubular, cystic, and folded blades, during green tides in the Yellow Sea in response to environmental changes (Zhang et al., 2013).

The morphology of *U*. *prolifera* changes with the environmental conditions (especially nutrient levels) during the drift from the SH to the NH. We hypothesized that the thalli of *U*. *prolifera* adapt to the environmental changes by the variation in morphology to enhance nutrient uptake in NH, an area with relatively less nutrients. To test this hypothesis, we investigated the SA:VOL of *U*. *prolifera* during the northward drift and analyzed the opportunist species from the perspective of morphological variation and associated physiological functional traits.

## 2 MATERIALS AND METHODS

### 2.1 Experimental design

**FIGURE 1:**
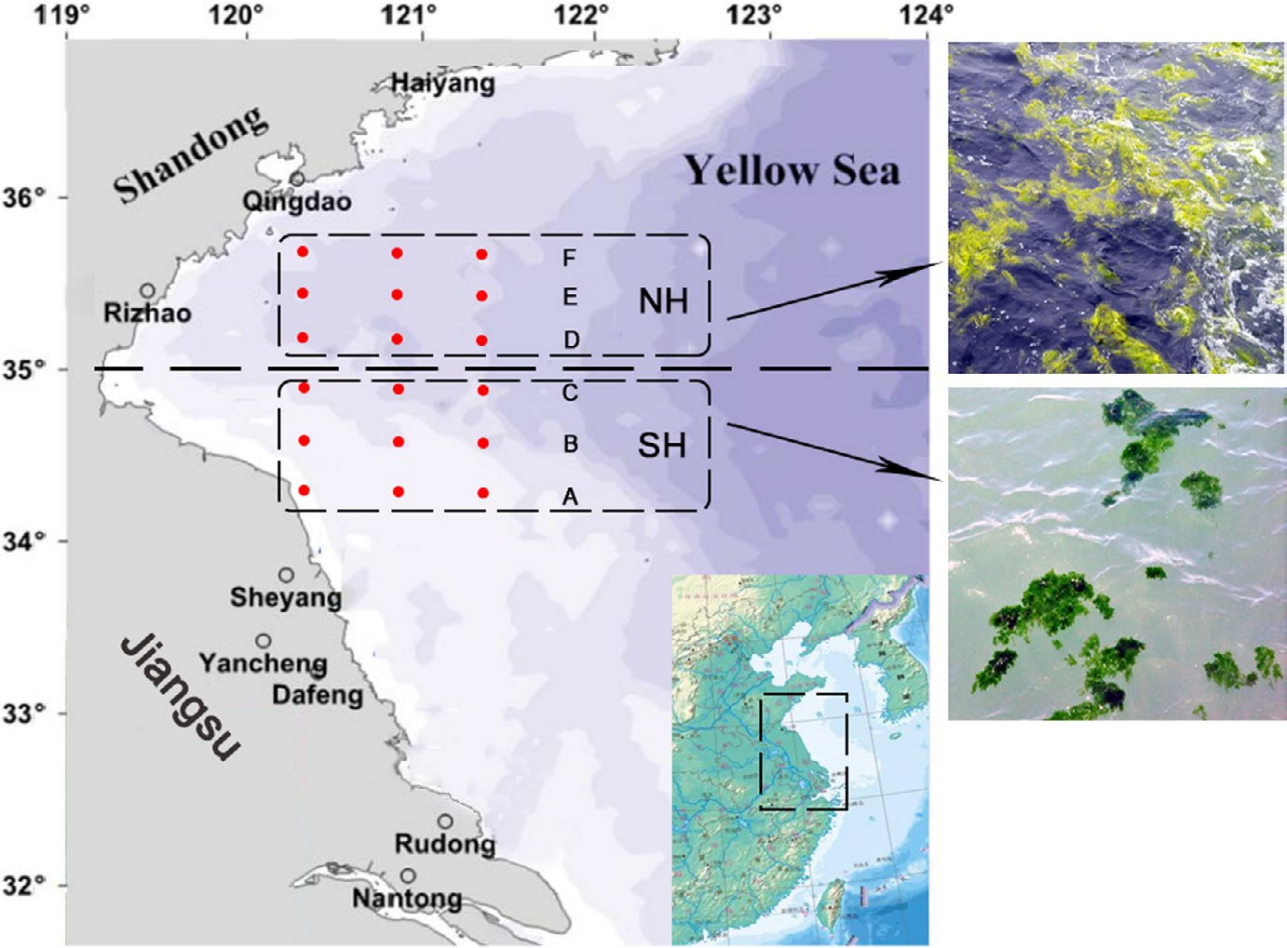
Locations of sampling sites in the southern area (SH; A/B/C) and northern area (NH; D/E/F) of Haizhou Bay (latitude around 35°N), Yellow Sea, China

Thalli of *U*. *prolifera* were collected from the SH (A/B/C) and NH (D/E/F) of Haizhou Bay in June 2018 during the *U*. *prolifera* green tides bloom period (Figure 1). Each sample line comprised three sites, and three samples of thalli were collected randomly at each site. The samples were cleaned with a brush, and sterile seawater was used to clean the surfaces of the thalli. The collected thalli were used directly for analyzing the ratio of surface area to volume (SA:VOL) and for measuring chlorophyll fluorescence parameters and reproductive allocation (RA) ratio in situ. The thalli were then stored in a freezer (SANYO, Japan) at −80°C before analysis of biochemical parameters. Our in‐situ observation data (lines A and B in Figure 1) showed that filamentous thalli were the dominant morphology, representing over 95% of the samples. With the northward drift, the filamentous thalli gradually changed to the tubular thalli. The filamentous thalli accounted for nearly 70% in sample line C and about 45% in sample line D. The tubular thalli were the dominant morphology in sample line F at over 90%.

### 2.2 Morphological observations

Samples of *U*. *prolifera* were photographed and observed for morphological characteristics of thalli such as the color, number, and shape of branches. Then, the thalli were sheared into 2‐mm slices to observe cellular details using a light microscope (RVL‐100‐G, ECHO, USA). The main branches of samples from SH and NH were cut into 400‐mm lengths to calculate the SA:VOL. The surface area was calculated using ImageJ (National Institutes of Health, USA) and HTPheno (IPK, Germany; Macinnis‐Ng et al., 2005; Ralph et al., 1998). Volume was calculated by a volumetric cylinder using water.

### 2.3 Relative growth rate

Relative growth rate (RGR) of *U*. *prolifera* was tested in situ over a 7‐day period. Samples were cultured in a mesocosm experiment. The fresh weight was obtained, and the RGR (%/day) was calculated as follows (Wu et al., 2018):

(1) $$RGR=\frac{\ln W_{2}‐\ln W_{1}}{T_{2}‐T_{1}}\times 100,$$

where W_1_ and W_2_ represent the fresh weight of samples at days T_1_ and T_2_, respectively.

### 2.4 The measurement of chlorophyll fluorescence parameters

Photosynthetic performance of *U*. *prolifera* was measured using a Dual‐PAM‐100 fluorometer (Walz, Germany). Before the experiments, the thalli were incubated in the dark for 20 min to ensure reliability of the data. The fluorometer settings followed those used in a previous study (Zhao et al., 2016). The induction, recovery curve routine, and repetitive application of saturation pulses were used to measure chlorophyll fluorescence parameters at room temperature. The parameter *F*_v_/*F*_m_ represented the photosystem II (PSII) maximum quantum yield in the thalli, and Y(II) represented the PSII actual quantum yield. The parameters *F*_v_/*F*_m_ and Y(II) were measured to evaluate the state of *Ulva prolifera*. *F_0_* was the minimal fluorescence after dark acclimation, and *F*_m_ was the maximal fluorescence after saturation flashes in the dark‐acclimatized sample. *F*_v_/*F*_m_ = (*F_m_* − *F_0_*)/*F_m_* and Y(II) = (*F_m_^’^*−*F_0_^’^*)/*F_m_’* (Maxwell & Johnson, 2000).

The thalli were exposed to a light intensity gradient (PAR: 0, 25, 40, 56, 73, 129, 169, 276, 342, 534, 828, 1354, and 1597 μmol photons m^−2^ s^−1^) to measure the rapid light–response curves (RLCs; Zhao et al., 2016). The photosynthetic capacity of the thalli was reflected by the RLCs. The parameters for photosynthetic rate in the light‐limited region of the RLCs (α) and the relative electron transport rate (rETR) were obtained by fitting Platt's empirical equation (Platt et al., 1980):

(2) $$P=P_{m}\times \left(1‐e^{‐\alpha \times PAR/P_{m}}\right)\times e^{‐\beta \times PAR/P_{m}},$$

where *P* is the rETR, Pm is the rETR_max_, *α* is the initial slope, and *β* is the photoinhibition parameter.

### 2.5 Analysis of biochemical parameters

Samples of the frozen thalli were weighed (50–100 g) and dried in a drying oven (DHG‐9145A, Yiheng, China) at 60°C for 12 h. Concentrations of nitrogen and phosphorus were quantified by inductively coupled plasma–optical emission spectrometry (5100 ICP‐OES, Agilent Technologies Inc.). The tissue carbon contents were measured using an autoanalyzer (AutoAnalyzer 3, Seal Analytical). Crude protein was measured using the Kjeldahl method (Wiedemair et al., 2019). Cellulose content is an indicator of the degree of mechanical tissue development of plant cells. Cellulose is a major component of crude fiber; this was measured using the Van method (Soest, 1990). Crude lipid was measured using the Soxhlet extraction method (McNichol et al., 2012).

### 2.6 Analysis of reproductive allocation

Thalli were cleaned with a brush, washed with sterile seawater, and placed on glass slides. The cells were observed using a light microscope (RVL‐100‐G, ECHO). The number of germ cells and the number of vegetative cells were counted to calculate RA (%) (Wang et al., 2019).

(3) $$\text{RA}=\frac{N_{g}}{N_{v}}\times 100$$

Here, *N_g_* is the number of germ cells and *N_v_* is the number of vegetative cells.

### 2.7 Statistical analyses

The data presented are the means (±*SD*) of three independent experiments. The data were initially examined for homogeneity using Levene's test and for normality using the Shapiro–Wilk test. Statistical significance was determined for *P*‐values less than 0.05. Samples of SA:VOL and RGR between six sampling lines were analyzed using one‐way analysis of variance (ANOVA), whereas samples of *F*_v_/*F*_m_, Y(II), tissue C, N, and P contents, crude protein, crude lipid, crude fiber, and RA between SH and NH were analyzed using Student's *t*‐test. These data analyses were performed using IBM SPSS Statistics for Windows 22.0 software (IBM Corporation) and graphically visualized using Origin software (OriginLab). The relationship between physiological functional traits and environmental factors were calculated using redundancy analysis in Canoco 5.0 by selecting analysis type “constrained ordination of species with selection of environmental variables.” Pearson correlation analyses between physiological functional traits and environmental factors were performed and visualized using Origin software. Collinearity diagnostics of linear regression and the ridge regression were performed using IBM SPSS Statistics. The PLS‐SEM was used to analyze the possible influential pathways of SA:VOL in physiological functional traits by SmartPLS 3.3 (SmartPLS GmbH). The environmental data referred to previous studies (Guan et al., 2021; Wang et al., 2018; Zhang, Su, et al., 2020).

## 3 RESULTS

### 3.1 Morphological observations

**FIGURE 2:**
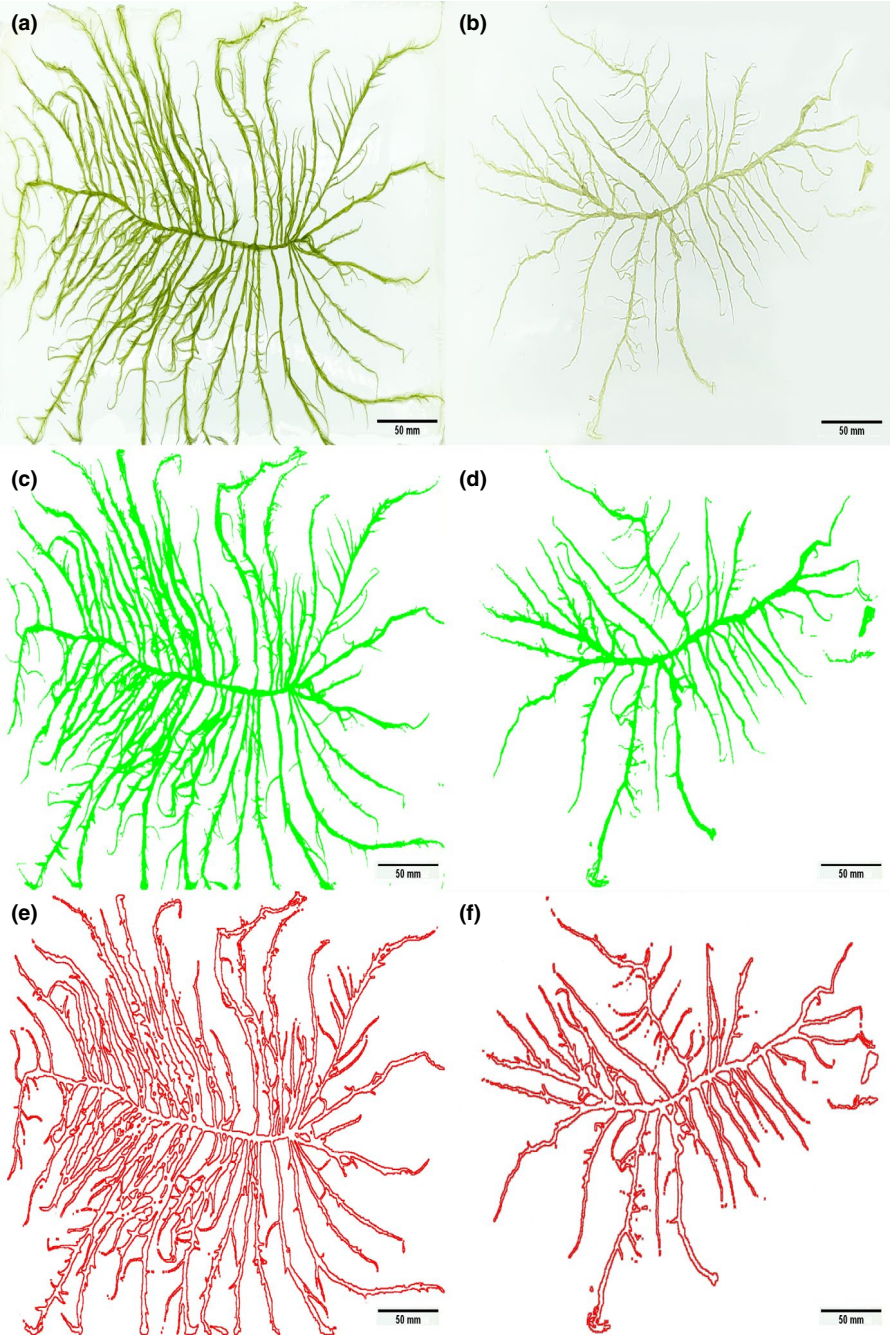
Morphology of *Ulva prolifera* thalli from the southern area (SH) and northern area (NH) of Haizhou Bay, Yellow Sea. (a) Morphology of thalli sampled from the SH. (b) Morphology of thalli sampled from the NH. (c) and (e) The surface areas of thalli in SH were calculated by ImageJ. (d) and (f) The surface areas of thalli in NH were calculated by ImageJ. Bars = 50 mm

Figure 2 shows the *U*. *prolifera* thallus morphology in the SH (Figure 2a,c,e) and the NH (Figure 2b,d,f). Thalli in the SH were dark green and comprised a long filamentous thallus with multiple short branches (Figure 2a). Thalli in the NH were light green, hollow tubular, and the branches were coiled together (Figure 2b).

**FIGURE 3:**
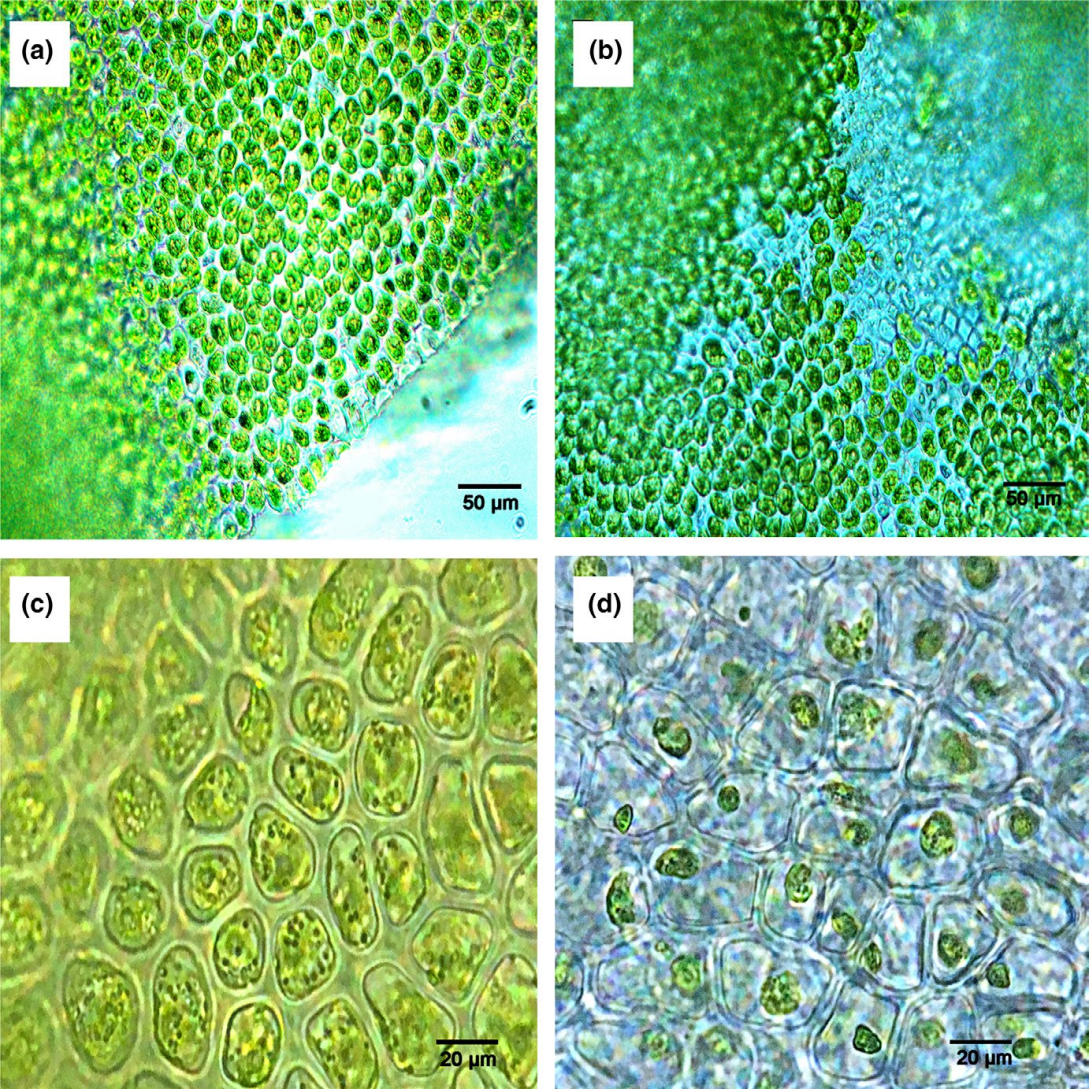
Morphological variation of *Ulva prolifera* thalli from the southern area (SH) and northern area (NH) of Haizhou Bay, Yellow Sea. Cellular details of thalli from the SH (bars: a, 50 µm; c, 20 µm) and the NH (bars: b, 50 µm; d, 20 µm)

Microscopic examination revealed that thalli in the SH contained numerous germ cells (Figure 3a,c). In thalli from the NH, spores and gametes had been released from the germ cells, while organelles were clumped on one side of the cells (Figure 3b,d).

### 3.2 The ratio of surface area to volume

**FIGURE 4:**
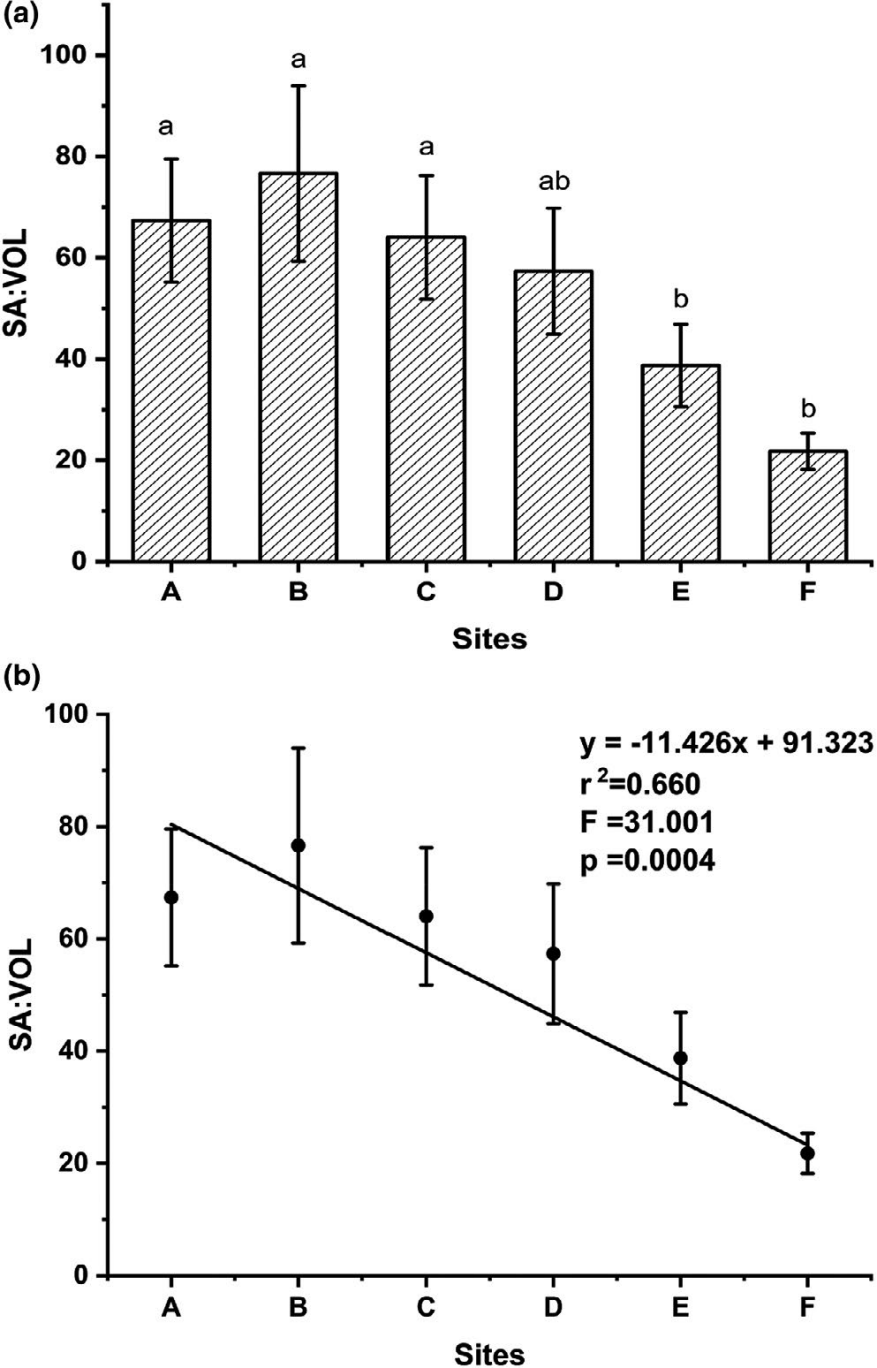
The values of SA:VOL of *Ulva prolifera* thalli from the southern area (SH; a–c) and northern area (NH; d–f) of Haizhou Bay, Yellow Sea. (a) SA:VOL of thalli. Values are means ± SD (*n* = 9). Different letters above bars indicate a significant difference (one‐way ANOVA, *p* < .05). (b) Relationship of mean (±SD) surface area to volume ratio (SA:VOL) of *Ulva prolifera* thalli from the sampling lines (a–f). Solid lines are the regression fits from the six sampling lines (*p* < .05; *n* = 9)

Values of SA:VOL ranged from 76.6 at sampling line B to 21.8 at sampling line F. Although the values in sampling line B were higher than in other sampling lines, SA:VOL tended to decline from SH to NH, as indicated by a significant negative relationship with latitude (Figure 4).

### 3.3 Results of relative growth rate

**FIGURE 5:**
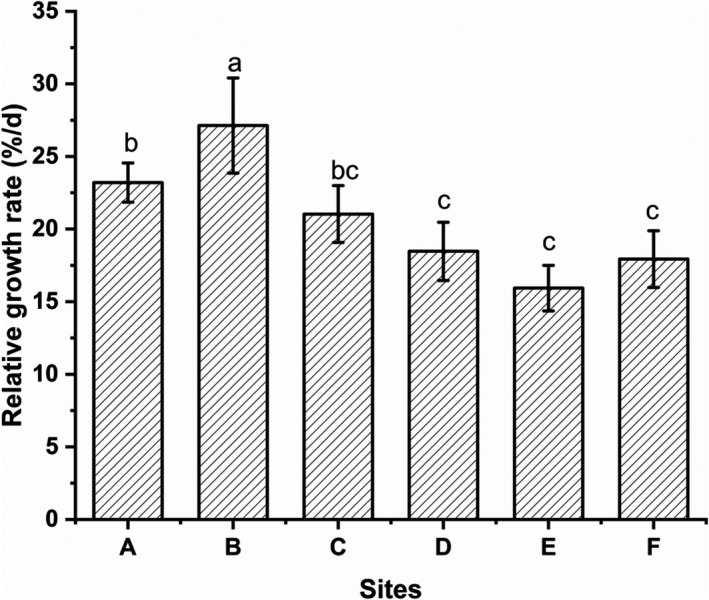
The values of RGR of *Ulva prolifera* thalli from the southern area (SH; a–c) and northern area (NH; d–f) of Haizhou Bay, Yellow Sea. Values are means ± SD (*n* = 9). Different letters above bars indicate a significant difference (one‐way ANOVA, *p* < .05)

The results of RGR showed a temporary increase and then a decreasing tendency during the drift northward. The RGR of *U*. *prolifera* was 27.13 ± 3.28%/d in sampling line B, which was significantly higher than in other sampling lines. The results in sampling line E showed the lowest value, 15.93 ± 1.57%/d, compared to other sampling lines (Figure 5).

### 3.4 Results of chlorophyll fluorescence parameters

**FIGURE 6:**
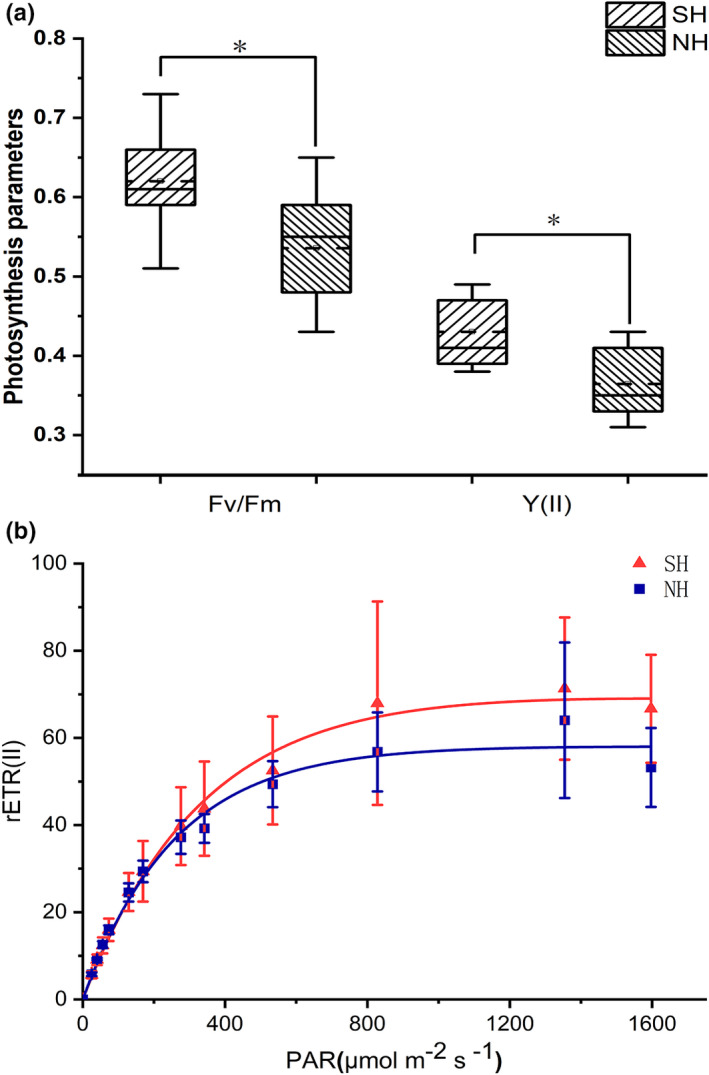
Photosynthetic parameters of *Ulva prolifera* thalli from the southern area (SH) and northern area (NH) of Haizhou Bay, Yellow Sea. (a) Box‐plot of *F_v_*/*F_m_* and Y(II). Values are means ± SD (*n* = 27). *Significant correlation at the 0.05 level. (b) Rapid light–response curves of the mean relative electron transport rate (rETR) versus photosynthetically active radiation (PAR) of *Ulva prolifera* thalli from the southern area (SH) and northern area (NH) of Haizhou Bay, Yellow Sea. Values are means ± SD (*n* = 27)

The values of *F*_v_/*F*_m_ and Y(II) for the SH thalli were higher than those of thalli from the NH, and *F*_v_/*F*_m_ and Y(II) showed significant differences between SH and NH (Student's *t*‐test, *p* < .05; Figure 6a). The values of *F*_v_/*F*_m_ and Y(II) for the SH thalli ranged from 0.51 to 0.73 and 0.38 to 0.49, respectively, while the values of *F*_v_/*F*_m_ from the NH ranged from 0.43 to 0.65 and 0.31 to 0.43, respectively (Figure 6a).

The RLCs increased linearly at light intensities that limited the photosynthetic capacity of the thalli. The plateau of the curve for thalli from the SH was higher than that for thalli from the NH (Figure 6b). The rETR_max_ and minimum saturating irradiance (*E*_k_) were significantly higher in the SH thalli compared with those of the NH thalli (Student's *t*‐test, *p* < .05, Table 1).

**TABLE 1:** Mean parameters for the rapid light–response curves of *Ulva prolifera* thalli from the southern area (SH) and northern area (NH) of Haizhou Bay, Yellow Sea. Values are means ± SD (*n* = 27). Different superscript letters within a column indicate significant differences (Student's *t*‐test, *p* < .05)

States	rETRmax	*α*	*β*	E_k_
SH	72.35 ± 10.87^a^	.21 ± .01^a^	.01 ± .00^a^	337.91 ± 68.69^a^
NH	58.56 ± 8.71^b^	.22 ± .02^a^	.01 ± .00^a^	260.76 ± 58.19^b^

### 3.5 Results for biochemical parameters

**FIGURE 7:**
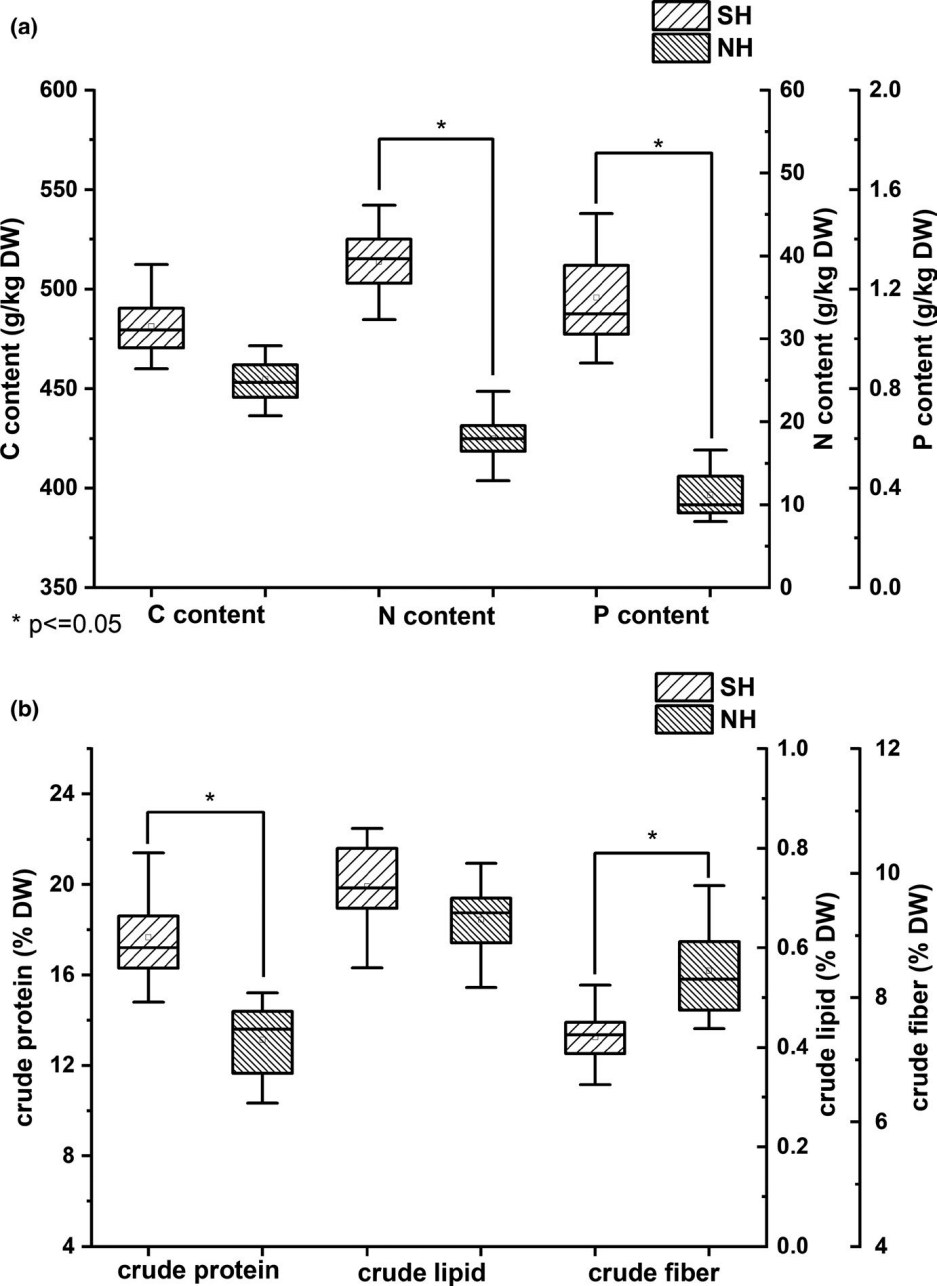
Biochemical parameters of *Ulva prolifera* thalli from the southern area (SH) and northern area (NH) of Haizhou Bay, Yellow Sea. (a) Box‐plots of tissue elemental analysis (C, N, and P). Values are means ± SD (*n* = 27). *Significant correlation at the 0.05 level. (b) Box‐plots of crude protein, crude fiber, and crude lipid. Values are means ± SD (*n* = 27). *Significant correlation at the .05 level

The tissue nitrogen and phosphorus contents in SH thalli were significantly higher than those of thalli in NH (Student's *t*‐test, *p* < .05, Figure 7a). The content of carbon showed no significant difference between SH and NH thalli (Student's *t*‐test, *p* > .05, Figure 7a).

The crude protein content of thalli from the SH was significantly higher than that of NH thalli (Student's *t*‐test, *p* < .05, Figure 7b). The crude fiber content of SH thalli was significantly lower than that of NH thalli (Student's *t*‐test, *p* < .05, Figure 7b). Although the lipid content of SH thalli was lower than that of NH thalli, the difference was not significant (Student's *t*‐test, *p* > .05, Figure 7b).

### 3.6 Reproductive allocation

**FIGURE 8:**
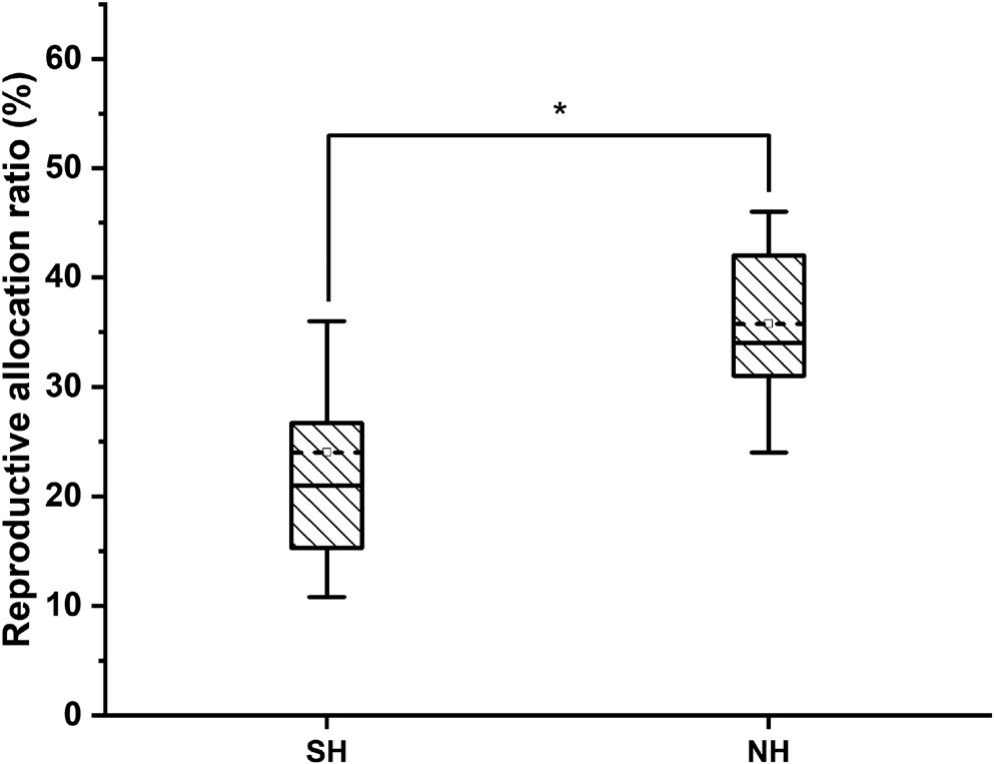
Box‐plots of reproductive allocation ratio (RA) of *Ulva prolifera* thalli collected from the southern area (SH) and northern area (NH) of Haizhou Bay, Yellow Sea. Values are means ± SD (*n* = 27). Different letters above boxes indicate a significant difference (Student's *t*‐test, *p* < .05)

The ratio of RA in NH thalli was significantly higher than that in SH thalli (Student's *t*‐test, *p* < .05, Figure 8). The values of RA for the SH thalli ranged from 10.8% to 45.5%, while the values of RA from the NH ranged from 17.2% to 58.0% (Figure 8).

### 3.7 Relationships between environmental and physiological parameters

**FIGURE 9:**
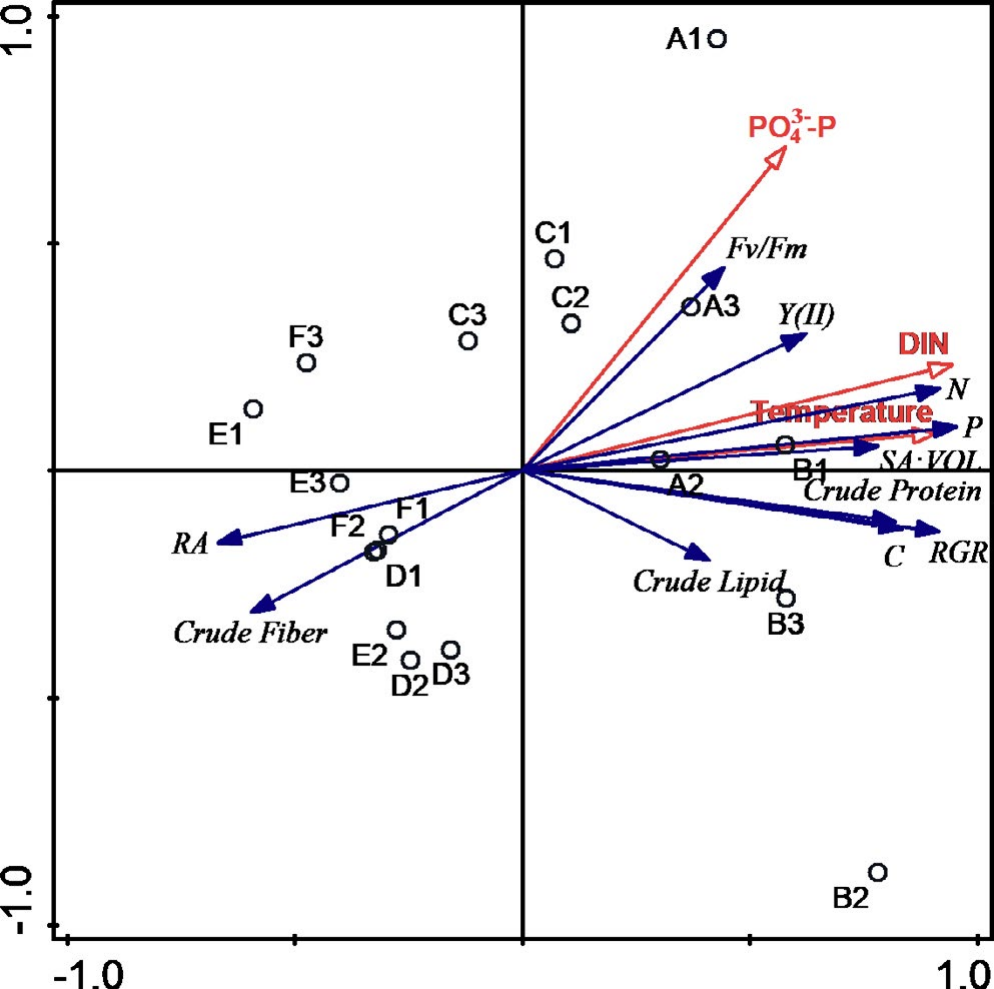
Redundancy analysis based on different environmental and physiological parameters of *Ulva prolifera* (*n* = 3 individuals per site) at the six study lines. Red arrows show environmental factors, and blue arrows show physiological functions of *U*.* prolifera*

Redundancy analysis reflected the influence of environment parameters on physiological indices, where the explanatory variables accounted for 68.7% of the variance. DIN, temperature, and PO_4_^3−^‐P were related to physiological functions of *U*. *prolifera*, and DIN showed the most significant relationship with biochemical parameters of *U*. *prolifera* (*p* = .002 < .05, Figure 9).

**FIGURE 10:**
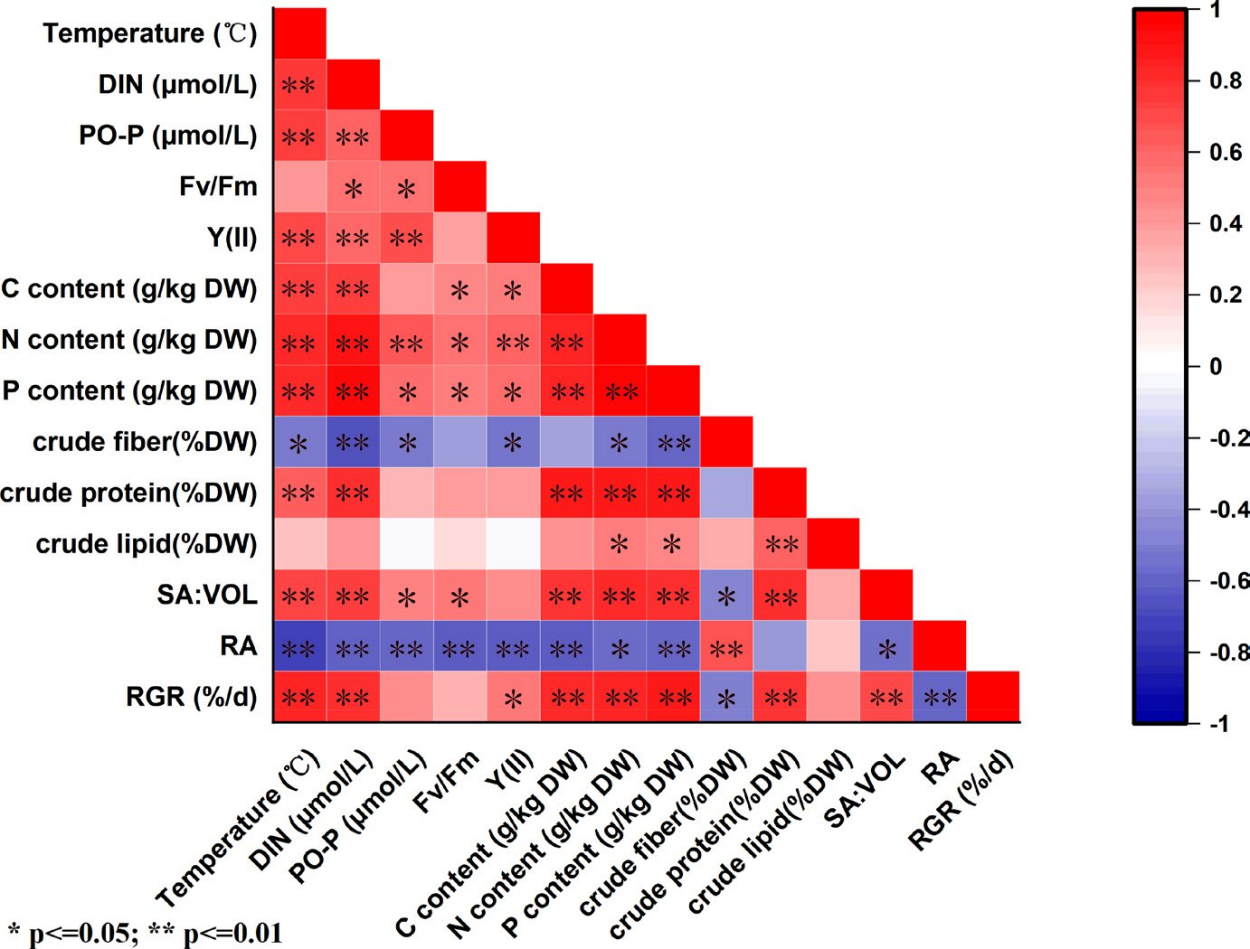
Correlation analysis between environmental factors and physiological parameters of *Ulva prolifera*. Different colors indicate different levels of the Pearson correlation coefficient. *Significant correlation at the .05 level. **Significant correlation at the .01 level

Figure 10 shows that SA:VOL of *U*. *prolifera* was significantly positively correlated with temperature, DIN, PO_4_^3−^‐P, Fv/Fm, tissue C, N, and P contents, crude protein, and RGR, and significantly negatively correlated with crude fiber and RA (*p* < .05, Figure 10). The correlation coefficients for N content, crude protein, and P and C contents were 0.831, 0.819, 0.818, and 0.790, respectively, reflecting the most significant relationships with SA:VOL (Figure 10). RA was significantly negatively correlated with temperature, DIN, PO_4_^3−^‐P, *F*_v_/*F*_m_, Y(II), tissue C, N, and P contents, crude protein, and RGR, and significantly positively correlated with crude fiber (*p* < .05, Figure 10). RGR had the strongest relationship with P content. In addition, crude fiber exhibited negative significant correlations with N and P contents (*p* < .05, Figure 10).

### 3.8 Physiological functional traits explain morphological variation of *Ulva prolifera*

**FIGURE 11:**
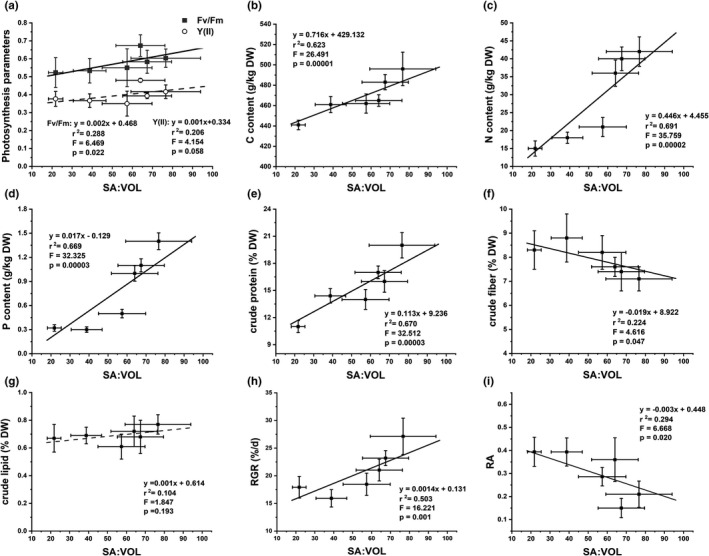
Relationships between mean (±*SD*) photosynthesis parameters (a), tissue of C (b), N (c), P (d) content, crude fiber (e), crude protein (f), crude lipid (g), RGR (h), and RA (i), and SA:VOL at each of the six study lines. Solid lines are the regression fits to data from study sites (*p* < .05, *n* = 9)

Individual parameters exhibited variable relationships with SA:VOL (Figure 11). Unitary linear regression was used to analyze the relationship between the independent variable (SA:VOL) and dependent variables (photosynthesis parameters, tissue of C, N, P contents, crude fiber, crude protein, crude lipid, RGR, and RA). RA and crude fiber had strong negative relationships with SA:VOL. In contrast, *F*_v_/*F*_m_, Y(II), tissue contents of C, N, P, crude protein, crude lipid, and RGR tended to increase with increasing values of SA:VOL, while Y(II) and crude lipid had insignificant negative relationships with SA:VOL (Figure 11).

**FIGURE 12:**
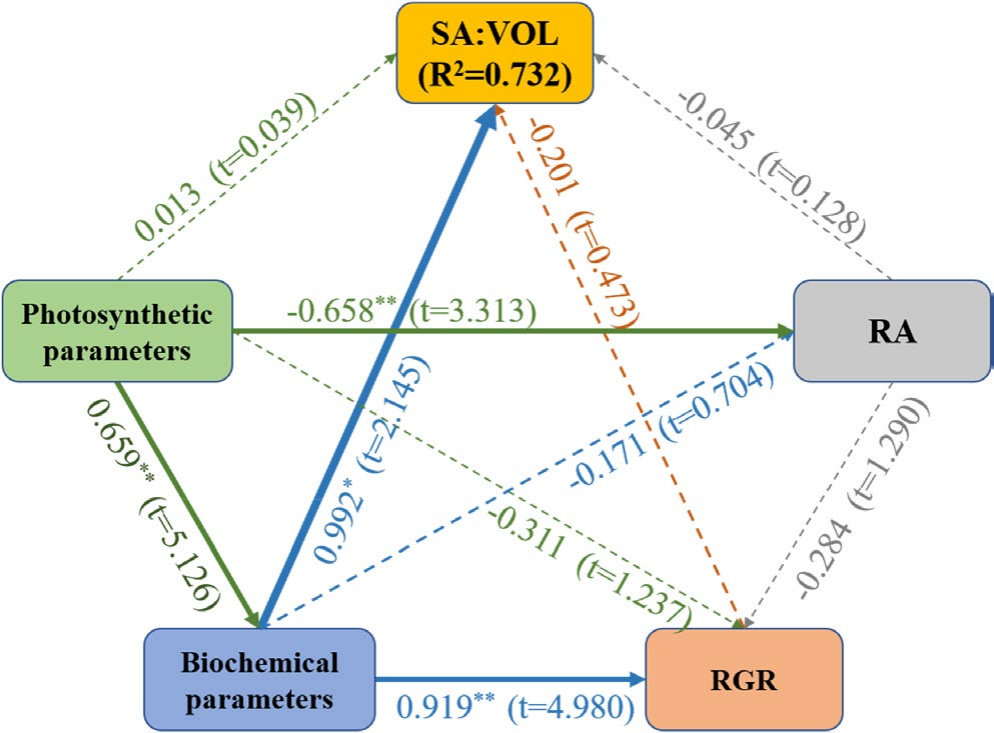
Pathway analysis using PLS‐SEM on influential factors of SA:VOL. Solid lines mean a significant influence. *Significant correlation at the .05 level. **Significant correlation at the .01 level

Collinearity diagnostics of linear regression were performed using IBM SPSS Statistics. The results identified the multicollinearity of these data (Tables S1, S2). The potential drivers for SA:VOL in *U*. *prolifera* of the Yellow Sea were investigated by pathway analysis using PLS‐SEM (Figure 12). Biochemical parameters and photosynthetic parameters had positive effects on SA:VOL with respective path coefficients of 0.992 and 0.013, whereas RGR and RA had negative effects on SA:VOL with respective path coefficients of −0.201 and −0.045. The explanatory variables accounted for 73.2% of SA:VOL. The PLS‐SEM analysis demonstrated that the biochemical parameters exerted a stronger impact on SA:VOL (Figure 12).

Biochemical parameters were further analyzed by the ridge regression method (Figure S1). The value of k was 0.8. The results showed that the explanation of six independent variables for SA:VOL was 71.2% (Table 2). The tissue contents of C, N, P, and crude protein changed significantly in the ridge regression analysis, and both of these indexes had a positive influence on SA:VOL. Crude fiber and crude lipid were not significantly changed in the ridge regression analysis. We obtained the following equation:

$$\begin{array}{c} \text{SA:VOL}=‐52.300+0.190*C+0.334*N+7.557*P‐2.357*\text{crude}\;\text{fiber}\\ +1.360*\text{crude}\;\text{protein}‐0.312*\text{crude}\;\text{lipid}. \end{array}$$

**TABLE 2:** Results of ridge regression analysis

	*B*	*SE*	*Β*	*t*	*p*	*R*²	*F*
Constant	−52.3	44.314	–	−1.18	.263	.712	*F* = 4.532 *p* = .015
C	0.19	0.077	.173	2.489	.030*		
N	0.334	0.095	.179	3.511	.005**		
P	7.557	2.119	.153	3.566	.004**		
Crude fiber	−2.357	1.876	−.094	−1.257	.235		
Crude protein	1.36	0.411	.189	3.306	.007**		
Crude lipid	−0.312	17.271	−.001	−0.018	.986		

Note

Dependent variable: SA:VOL.

* *p* < .05, **p < .01.

## 4 DISCUSSION

*Ulva prolifera* displayed distinct morphological differences during the drift northward. Thalli were dark green and comprised a long filamentous thallus with multiple short branches in SH, while being light green, hollow tubular, and with coiled branches in NH (Figure 2). This study tested the hypothesis that the thalli of *U*. *prolifera* adapted to the environmental changes via morphological variation to enhance nutrient uptake ability in the NH, an area with relatively less nutrients. Thus, we investigated the SA:VOL and physiological functional traits of *U*. *prolifera* with variation in morphology in Haizhou Bay. The value of SA:VOL decreased from the SH to NH of Haizhou Bay, negating the adaptation hypothesis. The physiological results suggested that functional traits affected morphological variation of *U*. *prolifera* in different environmental conditions during the drifting of green tides, and the trade‐off between growth and reproduction was the primary driving factor for this pattern.

### 4.1 The hypothesis concerning morphological variation of *Ulva prolifera*

Previous study identified *Ulva* as a developmentally plastic genus regarding morphology (Wichard et al., 2015). In addition, many studies have indicated that morphological changes in macroalgae are usually adaptations to changing environmental factors (Blain et al., 2020; Valiela et al., 1997). The adaptability to the environment is an important characteristic of *U*. *prolifera* (Gao et al., 2016; Shen et al., 2019). In the laboratory, *U*. *prolifera* acclimatizes to changes in seawater temperature and salinity by morphology‐driven physiological and biochemical variation; the thalli were in an aggregated form at lower temperature (20°C) and salinity (10) while being dispersed at higher temperature (25°C) and salinity (30) after 4 weeks of culture (Gao et al., 2016). A high SA:VOL ratio exhibited high rates of nutrient uptake for four intertidal seaweeds of Rhodophyceae and Phaeophyceae (Phillips & Hurd, 2004). *Ulva* may acclimate to changes in environmental conditions by varying its morphology after a relatively long time (Gao et al., 2016). Thus, we hypothesized that *U*. *prolifera* acclimatizing to the environment caused the SA:VOL in the thalli to have a negative relationship with nutrients.

However, the observed differences in morphology were in contrast to this hypothesis in the present study. The SA:VOL ratio of thalli from the SH was significantly higher than in algae from the NH (Figure 4), and SA:VOL had positive relationships with temperature and nutrients (Figure 10). Previous studies have found similar relationships between environmental changes and morphological variation. Kelp at the two lowest light sites had short stipes, while kelp at the mid‐to‐high light sites had longer stipes, suggesting that kelp preferentially put energy into the thallus and structural development rather than stipe elongation, which increases thallus SA:VOL, to increase irradiance in low‐light conditions (Blain et al., 2020). The growth environment of *U*. *prolifera* changed during the drift northward, where nutrient levels decreased, but nutrients were still above the minimum nutrients that limited *U*. *prolifera* growth in the north Yellow Sea (Zhang, Wang, et al., 2020). Thus, we speculated that morphological variation of *U*. *prolifera* was the result of regular growth, and the hypothesis of adapting to the environment did not apply to variation in morphology of *U*. *prolifera* in Hayzhou Bay. Instead, the results suggested that *U*. *prolifera* as an opportunist species was confronted with rapidly changing environmental factors in a short time during the northward drift in the Yellow Sea.

Environmental changes (DIN, temperature, and PO_4_^3−^‐P) also affected physiological parameters of *U*. *prolifera*. Nutrients and temperature were positively correlated with SA:VOL and negatively correlated with RA (Figure 9). In addition, the analysis showed that correlations between SA:VOL and environmental factors were lower than those between SA:VOL and physiological parameters (Figure 10). Thus, compared with environmental factors, SA:VOL of *U*. *prolifera* was more affected by variation in growth indices in Haizhou Bay. Temperature and nutrients were the main extrinsic controlling factors for morphology and RA variation. Therefore, the results suggest that it was not environmental changes but rather physiological functional trait variation of *U*. *prolifera* driving morphological differences in vivo.

### 4.2 Physiological functional traits affect morphological variation of *Ulva prolifera*

Previous studies have shown that the SA:VOL of macroalgae could predict photosynthetic activity and that macroalgae increased SA:VOL of thalli to increase exposure of photosynthetic tissues to light (Clark et al., 2018; Dromgoole, 1980; Enríquez et al., 1996; Miller et al., 2011). The *F*_v_/*F*_m_ and Y(II) values of the SH thalli were significantly higher than those of NH thalli (Figure 6a), and the result of RLCs showed photosynthetic activity of thalli in SH was higher than in NH (Figure 6b). At the same time, C, N, P, and crude protein contents of thalli from the SH were significantly higher than in NH thalli (Figure 7a), indicating that *U*. *prolifera* absorbed and stored the rich nutrients as an important material basis for growth in SH. Macroalgae like *U*. *prolifera* can rapidly absorb large amounts of nutrients in a short period of time to grow (Kamer et al., 2001; Li, 2015). Furthermore, a positive linear relationship existed between SA:VOL and photosynthesis parameters, similar to the relationship between SA:VOL and C, N, P, and crude protein contents (Figure 11). The PLS‐SEM showed that photosynthesis parameters had a highly positive effect on biochemical parameters, with a contribution coefficient of 0.659. The biochemical parameters had the greatest influence on SA:VOL, with a contribution coefficient of 0.992 (Figure 12). Therefore, the results suggested that a long filamentous thallus with multiple short branches of *U*. *prolifera* had higher photosynthetic activity in SH, as this facilitated allocation of energy to maximize the absorption and storage of nutrients.

The value of RA in NH was significantly higher than that of thalli in SH (Figure 8), and there was a negative relationship with SA:VOL (Figures 11 and 12). Previous studies have found that the explosive growth of biomass depends partly on rapid reproduction of *U*. *prolifera* (Gao et al., 2010; Li et al., 2014), and the formation of germ cells, especially sporulation, consumes large amounts of proteins, including tubulin, centrin, and cytoskeletal protein (Wang et al., 2016). The results of this study presented a similar phenomenon. The decreases in C, N, P, and crude protein contents in thalli from the NH suggested that proteins may be used to form large quantities of germ cells (Figure 7). Photosynthesis parameters had a highly negative effect on RA, with a contribution coefficient of −0.658 (Figure 12). Meanwhile, we observed that the photosynthetic activity of *U*. *prolifera* decreased gradually from SH to NH, and vegetative cells of *U*. *prolifera* formed into germ cells (Figures 3 and 6). The results were coherent with studies showing that the photosynthetic activity decreased rapidly when sporangia formed in *U*. *prolifera*, and the photosynthetic activity of vegetative cells was significantly higher than in sporangia (Gao et al., 2010; Wang et al., 2016; Zheng et al., 2018). A large reduction in vegetative cells may cause a decrease in photosynthetic activity of thalli. *U*. *prolifera* thalli with different colors could indicate that they are in different stages of sporangium formation (Lin et al., 2011). In this study, we found that the thalli in SH were dark green with low RA, whereas the thalli in NH were light green with high RA. Thus, the thallus color may reflect the extent of sporangium formation. Meanwhile, the vacuoles occupied most of the cell space, and the chloroplasts were pushed to the edges of the cells in the light green thalli (Lin et al., 2011). Similar results were found in this study. The thalli cells in NH showed organelles clumped on one side of the cells (Figure 3b,d). Therefore, RA was negatively associated with SA:VOL and photosynthetic activity of *U*. *prolifera*, as sporangia cells formed and thereby decreased photosynthetic activity and SA:VOL of thalli during northward drifting.

The values of RGR in SH were higher than those in NH, but RGR in SH and NH maintained relatively high values that were greater than 10% (Figure 5). The values of RGR showed an increasing and then decreasing trend during the northward drift (Figure 5) and were negatively correlated with RA (Figure 10). The growth rate and RGR were also consistent with the rate of biomass accumulation at the bloom (Wang et al., 2015). Previous study also showed that the biomass of *U*. *prolifera* maintained an increasing trend during the northward drift (Wang et al., 2015; Zhang et al., 2013). The thalli in SH had higher *F*_v_/*F*_m_, Y(II), C, N, P, and crude protein contents than NH thalli (Figures 6, 7). Biochemical parameters had highly positive effects on RGR, with a contribution coefficient of 0.919 (Figure 12). The correlation analysis showed that most of the growth indices were positively correlated with RGR (Figure 10). Thus, the results suggested that the growth of *U*. *prolifera* mainly contributed to the higher value of RGR.

The crude protein positively influenced SA:VOL according to PLS‐SEM and the ridge regression analysis. The results indicated that crude protein, especially some functional proteins, influenced SA:VOL of *U*. *prolifera* instead. Previous studies have found that proteins such as transcription factors play important roles in the ability of *U*. *prolifera* to respond to environmental changes via different morphologies. Wall‐associated receptor‐like kinase proteins served a vital role in cell elongation and were required for plant development (Lally et al., 2001). Brassinosteroids promoted Ovate Family Protein 1 to modulate plant architecture (Xiao et al., 2017). SIPRE2, one of the basic helix‐loop‐helix (bHLH) proteins, affected plant morphology (Zhu et al., 2017). However, it is unclear which proteins are responsible for morphological variation in *U*. *prolifera*. Thus, the specific functional proteins in *U*. *prolifera* need further investigation in future studies. Ultimately, the physiological functional traits may be the most important factors in the morphological variation of *U*. *prolifera* in different environmental conditions during the drift northward in the Yellow Sea.

## 5 CONCLUSIONS

This study focused on the importance of physiological functional traits, including growth and reproduction, in morphological variation of *U*. *prolifera*. The filamentous thalli with short, multiple branches in SH had higher growth rate, whereas the hollow and tubular thalli with coiled branches in NH had higher RA rates. The morphological variation led to a reduction in SA:VOL during the drift northward. Contrary to the original hypothesis, the results of the present study suggest that it was not environmental changes driving morphological differences. Further analysis showed that SA:VOL was correlated with physiology and biochemistry, suggesting that morphological variation was controlled by intrinsic functions of *U*. *prolifera* in Haizhou Bay. Thus, physiological functional traits affected morphological variation of *U*. *prolifera* in different environmental conditions during the drifting of green tides, indicating a trade‐off relationship between growth and reproduction during the drift northward.

## CONFLICT OF INTEREST

There are no conflicts of interest for all the authors including the implementation of research experiments and writing this article.

## AUTHOR CONTRIBUTION

**Ying Wang:** Conceptualization (equal); Data curation (equal); Methodology (equal); Writing – original draft (equal). **Chen Guan:** Data curation (equal); Formal analysis (equal); Investigation (equal); Visualization (equal); Writing – original draft (equal). **Xuexi Tang:** Conceptualization (equal); Methodology (equal). **Xinyu Zhao:** Investigation (equal); Methodology (equal). **Tongfei Qu:** Investigation (equal); Methodology (equal). **Yi Zhong:** Supervision (equal); Validation (equal). **Chengzong Hou:** Investigation (equal); Methodology (equal); Validation (equal). **Zhihao Lin:** Investigation (equal). **Jinhui Xu:** Validation (equal).

## Supporting information

Supplementary Material

## Data Availability

The data are available at the Dryad (https://doi.org/10.5061/dryad.w9ghx3fq5).
