# Supplementary material for: Physiological functional traits explain morphological variation of Ulva prolifera during the drifting of green tides
Source: Ecol Evol. 2022 Jan 17;12(1):e8504. doi: 10.1002/ece3.8504 (PMC8809434; doi:10.1002/ece3.8504)
Supplement: Supplementary file 1 — Supplementary Material [file ECE3-12-e8504-s001.docx]

**Table S1.** Coefficients of linear regression

| Model | | Unstandardized Coefficients | | Standardized Coefficients | t | Sig. | Collinearity Statistics | |
| --- | --- | --- | --- | --- | --- | --- | --- | --- |
|  |  | B | Std. Error | Beta |  |  | Tolerance | VIF |
| 1 | (Constant) | -64.576 | 283.866 |  | -.227 | .827 |  |  |
|  | Fv/Fm | 94.787 | 93.516 | .358 | 1.014 | .345 | .214 | 4.666 |
|  | Y(II) | -17.960 | 116.040 | -.046 | -.155 | .881 | .302 | 3.312 |
|  | C | -.065 | .729 | -.059 | -.089 | .932 | .061 | 16.310 |
|  | N | 1.293 | 2.684 | .693 | .482 | .645 | .013 | 77.329 |
|  | P | -16.134 | 102.481 | -.326 | -.157 | .879 | .006 | 160.561 |
|  | crude fiber(%DW) | 4.832 | 16.067 | .192 | .301 | .772 | .066 | 15.249 |
|  | crude protein(%DW) | 5.257 | 5.155 | .729 | 1.020 | .342 | .052 | 19.060 |
|  | crude lipid(%DW) | -152.425 | 150.622 | -.662 | -1.012 | .345 | .063 | 15.979 |
|  | RA | 71.023 | 119.096 | .360 | .596 | .570 | .074 | 13.595 |
|  | RGR | 202.078 | 274.610 | .396 | .736 | .486 | .093 | 10.800 |
| a. Dependent Variable: SA:VOL | | | | | | | | |

**Table S2.**  Collinearity diagnostics of linear regression

| Model | Dimension | Eigenvalue | Condition Index | Variance Proportions | | | | | | | | | | |
| --- | --- | --- | --- | --- | --- | --- | --- | --- | --- | --- | --- | --- | --- | --- |
|  |  |  |  | (Constant) | Fv/Fm | Y(II) | C | N | P | crude fiber(%DW) | crude protein(%DW) | crude lipid(%DW) | RA | RGR |
| 1 | 1 | 10.516 | 1.000 | .00 | .00 | .00 | .00 | .00 | .00 | .00 | .00 | .00 | .00 | .00 |
|  | 2 | .384 | 5.231 | .00 | .00 | .00 | .00 | .00 | .00 | .00 | .00 | .00 | .01 | .00 |
|  | 3 | .059 | 13.395 | .00 | .01 | .01 | .00 | .00 | .00 | .00 | .00 | .00 | .06 | .00 |
|  | 4 | .015 | 26.622 | .00 | .12 | .05 | .00 | .01 | .00 | .00 | .00 | .00 | .00 | .08 |
|  | 5 | .011 | 30.506 | .00 | .00 | .21 | .00 | .01 | .00 | .00 | .01 | .01 | .06 | .05 |
|  | 6 | .006 | 42.366 | .00 | .10 | .05 | .00 | .03 | .02 | .03 | .00 | .03 | .11 | .03 |
|  | 7 | .005 | 44.092 | .00 | .02 | .00 | .00 | .00 | .00 | .00 | .21 | .02 | .00 | .09 |
|  | 8 | .002 | 73.803 | .00 | .02 | .32 | .00 | .28 | .03 | .04 | .04 | .16 | .01 | .03 |
|  | 9 | .001 | 94.792 | .03 | .29 | .03 | .01 | .08 | .24 | .04 | .15 | .01 | .12 | .59 |
|  | 10 | .000 | 195.814 | .06 | .43 | .33 | .04 | .53 | .62 | .88 | .01 | .76 | .34 | .02 |
|  | 11 | 6.152E-5 | 413.438 | .91 | .01 | .00 | .96 | .05 | .08 | .00 | .57 | .01 | .30 | .12 |
| a. Dependent Variable: SA:VOL | | | | | | | | | | | | | | |


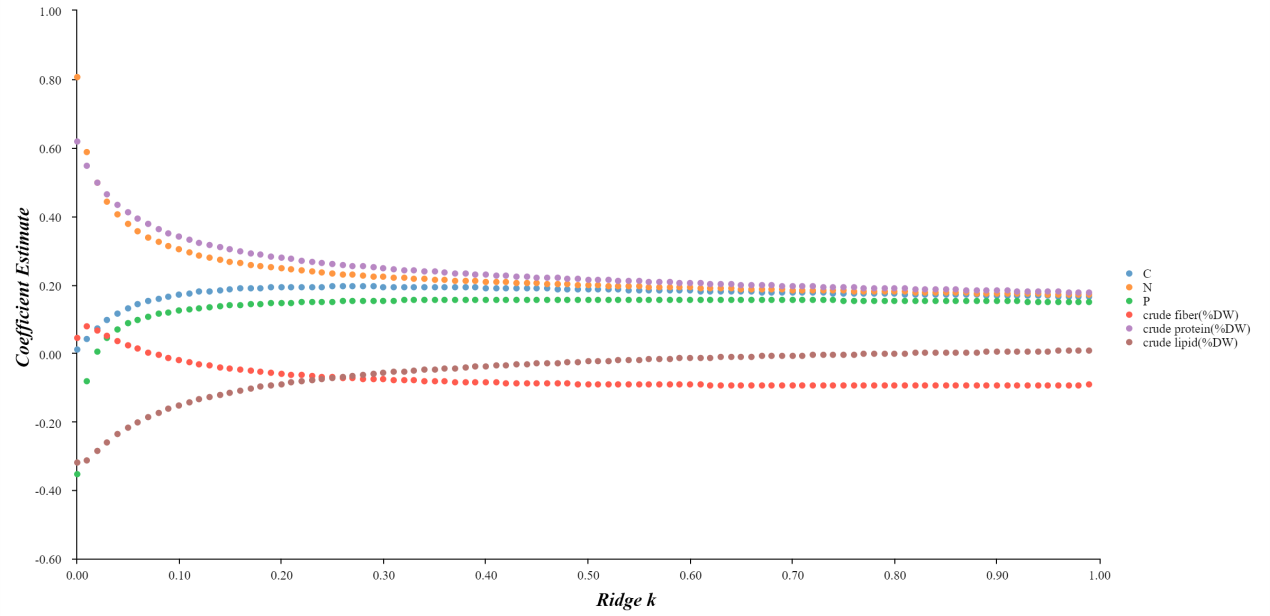


**Figure S1.** Ridge trace of six biochemical parameters
